# Extraction Components and Dyeing Effect of *Cotinus coggygria* Scop. in Water–Ethanol Systems

**DOI:** 10.3390/ma19040647

**Published:** 2026-02-07

**Authors:** Yuhe Liu, Zheng Xie, Yanan Tang, Zixin Dai, Liangjun Xia, Yunli Wang, Dan Sheng, Weilin Xu

**Affiliations:** 1 State Key Laboratory of New Textile Materials and Advanced Processing, Wuhan Textile University, Wuhan 430200, China; 18327086534@163.com (Y.L.); tangyanan1020@163.com (Y.T.); 15997619232@163.com (Z.D.); liangjun_xia@wtu.edu.cn (L.X.); weilin_xu0@163.com (W.X.); 2 School of Textile Science and Engineering, Wuhan Textile University, Wuhan 430200, China; 13125055229@163.com (Z.X.); ylwang@wtu.edu.cn (Y.W.)

**Keywords:** *Cotinus coggygria* Scop., pigment composition, water–ethanol system, antibacterial properties

## Abstract

As a highly representative traditional plant dye, the pigment composition of *Cotinus coggygria* Scop. (CCS) and its dyeing mechanisms require further study. In this work, pigments were extracted from the trunk of CCS using environmentally friendly solvents, namely ethanol and water. A systematic analysis of the components in the CCS extract and the stripping solutions from dyed cotton fabrics was conducted using LC-MS and UV-Vis spectroscopy, enabling an exploration of how different solvents influence the dissolution of CCS pigments and their dyeing effects on cotton fibers. The results indicated that water is more effective for extracting flavonoid glycosides and proanthocyanidins, whereas ethanol demonstrated superior efficacy in extracting flavonols and flavonol glycosides. Additionally, the dyed samples exhibited significant antibacterial activity against *Staphylococcus aureus*. The water–ethanol extraction and dyeing technology developed in this study aligns with the principles of sustainable development in green chemistry, providing an environmentally friendly solution for the industrial application of CCS and offering substantial ecological and economic value.

## 1. Introduction

Environmental and health concerns have prompted a reduction in the use of synthetic dyes, with natural dyes emerging as a promising alternative [1,2]. Natural dyes are mainly derived from plants, and the wastewater generated during their production is low in toxicity and biodegradable [3]. Furthermore, certain dyes (e.g., *Artemisia argyi* and indigo) possess medicinal properties, including antibacterial and insect-repellent effects [4,5,6,7].

*Cotinus coggygria* Scop. (CCS), commonly known as the smoke tree, is a plant belonging to the *Anacardiaceae* family [8]. It is sporadically distributed across various countries in southeastern Europe and is primarily found in the southwestern and northern regions of China [9,10,11]. Historically, CCS has been an important source of yellow dye [12,13]. Research on ancient artifacts confirms its widespread use in textile dyeing [14]. Modern studies show that CCS extract not only has excellent dyeing properties but also exhibits anti-inflammatory, antimicrobial, and antioxidant effects [15,16,17]. The historical application of CCS reflects ancient human practices in the sustainable use of natural dyes, offering technical references for contemporary green chemistry and bio-based resource development.

Despite its long history as a dye, systematic studies on the molecular structure of CCS pigments, their dissolution, and the mechanisms underlying the dyeing process remain limited. Türkmen et al. studied traditional dyes in the eastern Mediterranean region of Turkey and found that CCS could produce colors ranging from yellow to olive-brown on wool when treated with different mordants, with a lightfastness rating ranging from 3.5 to 4.5 [18]. Valianou et al. identified eleven flavonoids from *Cotinus coggygria* heartwood and detected multiple components in post-Byzantine silk textiles, confirming the historical use of CCS as a yellow dye [19]. Thus, research on CCS pigments holds significant application implications.

In the extraction of natural dyes, the selection of solvents is a critical factor [20,21]. Ethanol is extensively utilized for the extraction of plant dyes due to its superior solubility and favorable safety profile [22]. In recent years, advancements in solvent recovery technologies have attracted increasing attention in water–ethanol systems [23,24]. These developments have significantly improved process sustainability and provided essential technical support for the green industrialization of natural dyes. Besides, the extraction efficiency of CCS varies significantly depending on the solvent system used [25,26]. In this context, a detailed investigation into the dissolution properties of the CCS pigment components in water–ethanol systems, as well as their binding mechanisms with fiber materials, not only addresses a gap in basic research within this field but also provides a theoretical foundation for optimizing the development of natural dyes.

Hence, this study investigated the dissolution characteristics of CCS pigment components in a water–ethanol mixed solvent system, analyzed their binding mechanism with natural cotton fibers, and established a systematic dyeing process framework through multi-parameter optimization. The findings not only provide a theoretical and technical foundation for the industrial application of CCS but also offer new insights and practical strategies for the sustainable development of natural dyes.

## 2. Materials and Methods

### 2.1. Materials

The following materials were used in this study: CCS sawdust from the trunk (Sichuan Province, China, flake form, ~1 cm × 2 cm); cotton fabric (40s yarn count, double plain weave, warp and weft density: 100 × 100 pcs/10 cm, Winner Medical Co., Ltd., Wuhan, China); methanol (AR grade); dichloromethane (AR grade); neutral detergent (Shanghai Kao Co., Ltd., Shanghai, China); aluminum potassium sulfate (KAl(SO_4_)_2_⋅12 H_2_O, AR grade); ethanol (AR grade); acetic acid (AR grade); sodium hydroxide (AR grade); artificial saliva (Dongguan Chuangfeng Automation Technology Co., Ltd., Dongguan, China); artificial sweat (Dongguan Chuangfeng Automation Technology Co., Ltd., Dongguan, China); *Staphylococcus aureus* (*S. aureus*; ATCC 29213, Gram-positive); *Escherichia coli* (*E. coli*; ATCC 25922, Gram-negative); and pure water.

### 2.2. Cotton Fabric Dyeing and Stripping

#### 2.2.1. Extraction Method

All extractions and dyeing procedures were performed using the HIF-24 infrared dyeing machine (Jingjiang Huaxia Technology Co., Ltd., Jingjiang, China). CCS was immersed in water or ethanol (10% *w*/*v*). The mixture was then heated to 80 °C at 2 °C/min, maintained at that temperature for 60 min, and then cooled to room temperature (25 °C). Subsequently, the mixture was filtered through a 300-mesh nylon mesh to obtain water and ethanol extracts.

#### 2.2.2. Cotton Fabric Dyeing

Water and ethanol CCS extracts were used to dye 1.0 g of cotton fabric at a bath ratio of 1:50. The mixture was heated to 80 °C at 2 °C/min in a HH-S4 water bath (Changzhou Guoyu Instrument Manufacturing Co., Ltd., Changzhou, China), maintained at that temperature for 40 min, and then cooled to room temperature (25 °C). The fabric was subsequently washed and dried.

#### 2.2.3. Dye Stripping and Concentration

The concentration was performed using a RE-2000A rotary evaporator (Shanghai Yarong Biochemical Instrument Factory, Shanghai, China) and an SHZ-D(III) vacuum pump (Wuhan Kerl Instrument Equipment Co., Ltd., Wuhan, China). The extract solution (10 mL) was concentrated to approximately 1 mL via evaporation at 65 °C and 850 Pa. The stripping solution (15 mL) was prepared by mixing acetic acid, methanol, and water in a 1:2:1 volume ratio. The dyed cotton fabric sample (0.5 g) was immersed in the stripping solution, and the color was stripped at 80 °C for 2 h. The fabric was then removed, washed, and air-dried. The stripping solution was concentrated under reduced pressure at 65 °C and 850 Pa to eliminate methanol, after which 15 mL of dichloromethane was added to remove moisture. Subsequently, the mixture was dried over anhydrous sodium sulfate and concentrated to 1 mL under reduced pressure.

### 2.3. Dyeing Methods

#### 2.3.1. Preparation of Extracts with Varying Ethanol Concentrations

Equal amounts of CCS were immersed in ethanol solutions of varying concentrations (0%, 10%, 20%, 30%, 40%, 50%, 60%, 70%, 80%, 90%, and 100%) at 10% *w*/*v*. The mixtures were heated to 70 °C at a rate of 2 °C/min, maintained at that temperature for 60 min, and then cooled to room temperature. Subsequently, they were filtered through a 300-mesh nylon mesh to obtain ethanol extracts.

#### 2.3.2. Stability of the CCS Extracts

To assess the thermal stability, the extracts were treated at 40, 60, 80, and 100 °C for 2 h and then rapidly cooled to room temperature. The acid–base stability was also evaluated by adjusting the pH of the extracts to various ranges (3–4, 5–6, 7–8, and 9–10) using anhydrous acetic acid or sodium hydroxide and allowed to stabilize for 2 h.

#### 2.3.3. Mordant Dyeing

Cotton fabrics were dyed with extracts of varying ethanol concentrations using pre-, meta-, and post-mordanting methods at a bath ratio of 1:50 with an alum concentration of 5 g/L. The mixtures were heated to 70 °C at 2 °C/min, maintained at that temperature for 40 min, and then cooled. The fabrics were subsequently washed and dried.

### 2.4. Characterization

#### 2.4.1. Color Characteristics

The K/S value, lightness (L*), red–green index (a*), and yellow–blue index (b*) of the dye were measured using a CS-820 color-matching instrument (color QC, version: 2.79, Hangzhou Caipu Technology Co., Ltd., Hangzhou, China). The transmission method was used to determine these values for the dye, whereas the reflectance method was employed to measure them for the fabric. The average values were calculated from measurements obtained at five different locations on the fabric. The K/S value was calculated using the following equations [27,28]:

(1) $$\mathrm{Stripping}\;\mathrm{percentage}=\frac{{\left(K/S\right)}_{0}-{\left(K/S\right)}_{1}}{{\left(K/S\right)}_{0}}$$

(2) $$\frac{K}{S}=\frac{{\left(1-R\right)}^{2}}{2R}$$

where (K/S)_0_ is the color strength before stripping, (K/S)_1_ is the color strength after stripping, *K* is the absorption coefficient, *S* is the scattering coefficient, and *R* is the reflectivity.

#### 2.4.2. UV–Vis Spectroscopy

The UV-3600 Plus UV–VIS–NIR spectrophotometer(UVProbe, version: 2.71, Shimadzu Co., Ltd., Kyoto, Japan) was used with 0–100% ethanol solutions as blanks. Correspondingly diluted extract and stripping solutions were prepared to ensure absorbance within an appropriate range. The spectra were recorded over a wavelength range of 200–800 nm with a scanning speed set to high, a sampling interval of 1.0, and each scan was repeated twice.

#### 2.4.3. LC-MS

The concentrated extract and stripping solutions were filtered through 0.22 μm microporous membranes and subsequently analyzed using an Agilent 1290II UHPLC-Q-TOF system (Agilent Technologies MassHunter Workstation Qualitative Analysis: Software, version: B.07.00 SP2, Agilent Technologies Inc., Santa Clara, CA, USA). A reversed-phase C18 column (Eclipse Plus C18, 100 mm × 4.6 mm, 1.8 μm) was used as the stationary phase for chromatographic separation, which was performed at a column temperature of 30 °C. The mobile phase consisted of water containing 0.1% (*v*/*v*) formic acid (A) and acetonitrile (B) at a flow rate of 0.25 mL/min. The following gradient elution was applied: 95% A from 0 to 0.3 min; 95%–5% A from 0.3 to 9.3 min; 5% A from 9.3 to 10.3 min; 5%–95% A from 10.3 to 10.5 min; and 95% A from 10.5 to 12 min. The injection volume was 2 µL. Mass spectra were obtained using electrospray ionization (ESI) in both positive (ESI+) and negative (ESI−) modes over a scan range of *m*/*z* 100–1000.

#### 2.4.4. Color Fastness

The dyed cotton fabric samples were evaluated for rubbing fastness (ISO 105-X12) [29] using a Y571N friction fastness tester (Nantong Hongda Testing Instrument Co., Ltd., Nantong, China) and wash fastness (ISO 105-C01) [30] using an SW-12A washing color fastness tester (Changzhou Dazhong Electronics Instruments Co., Ltd., Changzhou, China). All samples were graded using a gray color-change sample card (GB/T 250–2008) [31] for cotton fabric color fastness.

#### 2.4.5. Antibacterial Properties

The antibacterial activity of cotton fabrics dyed with water and ethanol extracts of CCS was evaluated according to the AATCC 100 standard [32]. The assessment was conducted against two representative bacterial strains: *Staphylococcus aureus* (ATCC 6538) and *Escherichia coli* (ATCC 25922).

Data plotting and statistical analysis were performed using OriginPro software (version 2025b, OriginLab Corporation, Northampton, MA, USA), Chemical structures were drawn using ChemDraw (version 20.0, PerkinElmer, Waltham, MA, USA).

## 3. Results and Discussion

### 3.1. Color Stripping Effect

**Figure 1:**
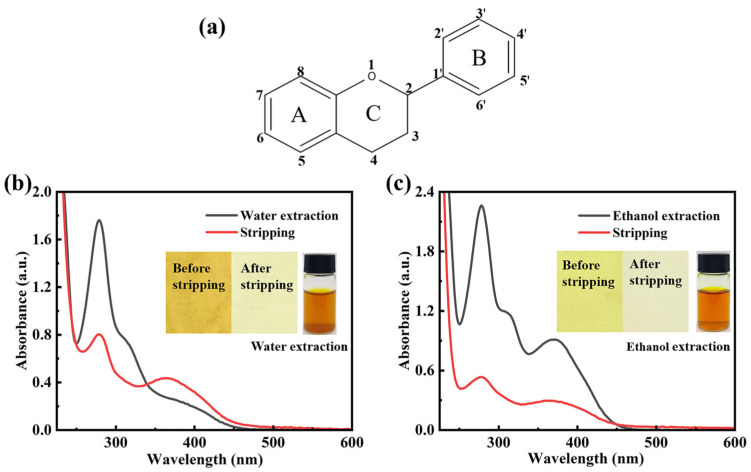
Flavonoid skeleton and UV–Vis absorption spectra of water and ethanol extracts and stripping solutions: (**a**) flavonoid skeleton; UV–Vis spectra of the (**b**) water extract and stripping solutions, and (**c**) ethanol extract and stripping solutions, along with corresponding images of the fabric before and after stripping.

Figure 1 shows that the water and ethanol extracts of CCS exhibit similar colors and hues. However, there is a significant difference in the color depth of the dyed cotton fabrics. The cotton fabric dyed with the CCS water extract exhibits a K/S value of 6.84, whereas the fabric dyed with the CCS ethanol extract has a K/S value of 1.22. When water is used as the solvent, the pigment components in CCS show a higher binding affinity for cotton fibers. To clarify the effect of solvents on the dissolution of CCS pigment components and the dyeing of cotton fibers, the color of the dyed cotton fabrics was stripped, and the pigment composition in the stripping solution was analyzed.

After stripping, the K/S value of cotton fabrics dyed with CCS water extract was 0.67, corresponding to a stripping percentage of 90.2%. For cotton fabrics dyed with CCS ethanol extract, the K/S value was 0.13, with a stripping percentage of 89.34%. The mixed solvent of methanol, acetic acid, and water (1:2:1) exhibited high stripping efficacy for both water and ethanol extracts of CCS-dyed fabrics, effectively removing the pigment.

The UV–Vis absorption spectra of the water and ethanol extracts and the stripping solutions were analyzed to determine whether any damage had occurred during the stripping process. For most flavonoids, the UV–Vis spectra exhibit two absorption bands: Band I (240–280 nm) and Band II (300–400 nm) [33]. Two absorption maxima at 350 nm (Band I, cinnamoyl system) and 267 nm (Band II, benzoyl system) are characteristic of the flavonoid skeleton (Figure 1a) [34,35]. Figure 1b,c show that the UV–Vis spectra of both the extracts and stripping solutions exhibit features characteristic of flavonoids. The characteristic absorption Band I in the range 350–385 nm is characteristic of flavonols [36]. The more pronounced Band I observed in the ethanol extract of CCS suggests that flavonols are more readily soluble in ethanol. Furthermore, the distinct Band I detected in the stripping solution from cotton fabrics dyed with the water extract confirms that the water extract also contains flavonol components and can bind with the cotton fibers.

### 3.2. Component Analysis

The effects of water and ethanol on the dissolution of CCS pigment components were clarified by analyzing the CCS water and ethanol extracts, as well as the stripping solutions from water- and ethanol-dyed fabrics, using LC-MS. Table 1 lists the compounds detected in both the CCS ethanol and water extracts based on LC-MS analysis. Twenty-one common pigment constituents were discovered, primarily flavonoids. These included flavanones (e.g., hesperetin and eriodictyol), flavone glycosides (such as kaempferol 3-rutinoside and kaempferol-3-O-glucorhamnoside), biflavonoids (bilobetin), and proanthocyanidins (including proanthocyanidin B2 and procyanidin B1), along with several other compounds (e.g., tropine and verbenalin) (Table 1). The substances isolated from the water extract included flavone glycosides (e.g., hesperetin 7-O-glucoside, vicenin I), flavones (e.g., dracorhodin, kuwanon G, engeletin), biflavonoids (amentoflavone, hinokiflavone), and other compounds (e.g., paederoside, kirenol, hypocrellin A) (Table 2). The different constituents in the CCS ethanol extract were flavonols (e.g., aromadendrin, quercitrin) and flavonol glycosides (e.g., quercitrin, laricitrin 3-O-glucoside). Further details are provided in the Supplementary Materials (Tables S1–S5). The results indicate that the solubility of CCS pigment is significantly influenced by both the properties of the solvent and the structure of the compounds. Flavonols exhibit good solubility in ethanol but limited solubility in water [37]. Flavonoid aglycones (such as quercetin and kaempferol) are hydrophobic owing to their structure [38]. Conversely, flavone glycosides are produced through the glycosylation of hydroxyl groups in flavones, such as kaempferol 3-rutinoside and vicenin-II, which increases their water solubility [39]. The number of glucosides and the binding sites also affect the solubility [40,41]. Flavonol glycosides exhibit low solubility in water due to their strong molecular planarity. Conversely, biflavonoid has a non-planar structure, which results in higher solubility in water compared to flavonol.

The compounds detected in the stripping solutions of CCS water and ethanol dyed fabrics were flavone glycosides (e.g., kaempferol 3-rutinoside, kaempferol-3-O-glucorhamnoside), biflavonoids (e.g., bilobetin), and proanthocyanidins (proanthocyanidin B2, procyanidin B1) (Table 3). Pigment components showed less difference. Furthermore, five substances were detected exclusively in the water stripping solution, including bilobetin, hesperetin, kirenol, hypocrellin A, and hinokiflavone (Table 4). One compound detected exclusively in the ethanol stripping solution was ombuoside. This suggests that the types of pigment components capable of binding to cotton fibers are minimally affected by the solvent. However, the dyed cotton fabrics exhibited considerable variation in color intensity. On the one hand, the differing solvents may lead to variations in the concentration of various compounds within the extracts. On the other hand, water has a better swelling effect on cotton fibers than ethanol [42,43], which enhances the binding of dye molecules to the fibers.

### 3.3. Effect of Ethanol Concentration on Pigment Components in CCS Extracts

**Figure 2:**
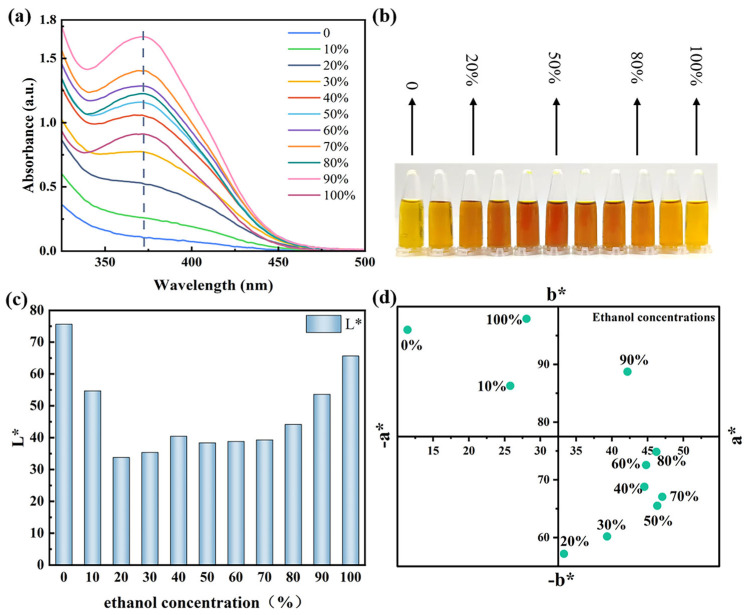
(**a**) UV absorption spectra of CCS extracts, (**b**) images of CCS extracts at different ethanol concentrations, (**c**) L* values, and (**d**) a* and b* values.

Ethanol and water exhibit different affinities for CCS pigments, and optimizing the water–ethanol system can enhance the extraction efficiency of pigments. The UV spectra of the CCS extracts at different ethanol concentrations consistently exhibit a peak at approximately 370 nm (Figure 2a), which can be attributed to the characteristic properties of flavonols. Moreover, the intensity of this peak increases with increasing ethanol concentration, further confirming that flavonols are more soluble in ethanol. As the ethanol concentration increased from 0% to 50%, the extract color transitioned from bright yellow to reddish-brown, with a corresponding gradual increase in color intensity. As the ethanol concentration further increased to 100%, the extract color shifted back from reddish-brown to bright yellow, and the color intensity gradually decreased (Figure 2b,c). Although the water and ethanol extract solutions of CCS pigment had similar colors, the chemical components were different.

The changes in color observed in the CCS extracts may be related to their composition and solubility. Furthermore, the ethanol concentration influences the release of different types of phenolic compounds and the total phenolic content of extracts [44]. As the ethanol concentration gradually increases, the solubility of ethanol-soluble compounds, such as flavonols, rises. Simultaneously, water-soluble pigments, including anthocyanins and flavonoid glycosides, continue to be released, leading to a gradual deepening of color in the extract. At ethanol concentrations above 50%, the solubility of water-soluble components decreases, while ethanol-soluble compounds gradually reach saturation. Consequently, the CCS extract becomes progressively lighter in color. Scarano et al. demonstrated that water–ethanol mixtures are more suitable for extracting phenolic compounds [21]. In this study, most of the identified pigments from CCS were phenolics, which is consistent with their findings. Therefore, for the extraction of CCS pigments, the 50% water–ethanol system demonstrated the highest efficiency.

### 3.4. Effects of Temperature and pH on the Pigments in CCS Extracts

**Figure 3:**
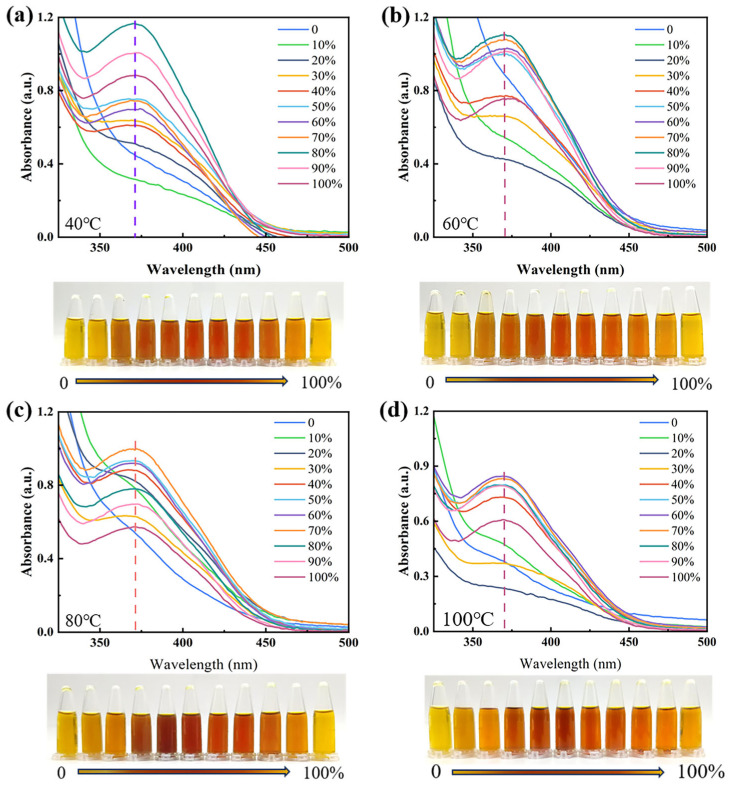
UV–Vis absorption spectra and images of CCS extracts at different ethanol concentrations following temperature treatment at (**a**) 40, (**b**) 60, (**c**) 80, and (**d**) 100 °C.

The CCS extracts were treated at 40, 60, 80, and 100 °C. The UV spectra consistently show a peak at approximately 370 nm (Figure 3). Both the peak position and shape remained unchanged, and the extracts showed no significant alteration in color. These results indicate that the pigment compositions maintained good structural stability from 40 °C to 100 °C.

**Figure 4:**
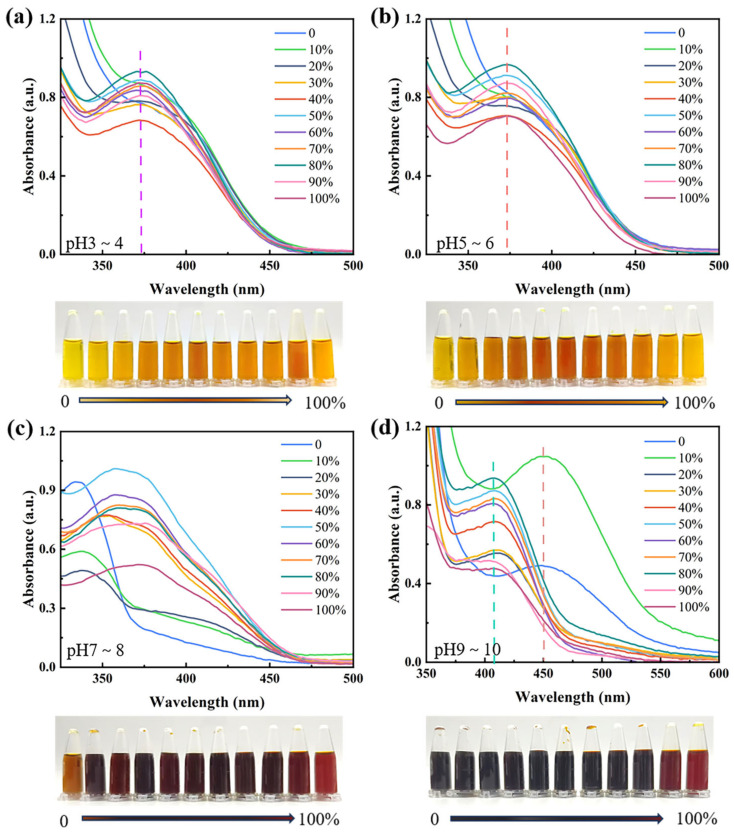
UV–Vis absorption spectra and images of CCS extracts at different ethanol concentrations after treatment under acidic and alkaline conditions: (**a**) pH 3~4, (**b**) pH 5~6, (**c**) pH 7~8, and (**d**) pH 9~10.

**Figure 5:**
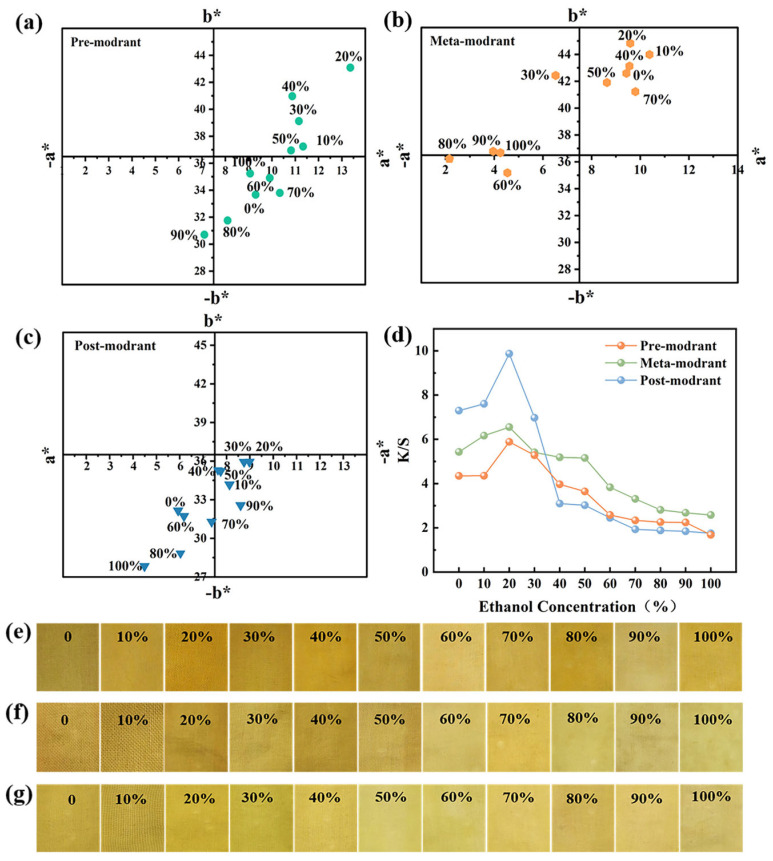
Dyeing effects of cotton fabrics using three mordanting methods at different ethanol concentrations: a* and b* values of (**a**) pre-mordanted, (**b**) meta-mordanted, and (**c**) post-mordanted cotton fabrics; K/S values of (**d**) mordanted, (**e**) pre-mordanted, (**f**) meta-mordanted, and (**g**) post-mordanted cotton fabrics.

**Figure 6:**
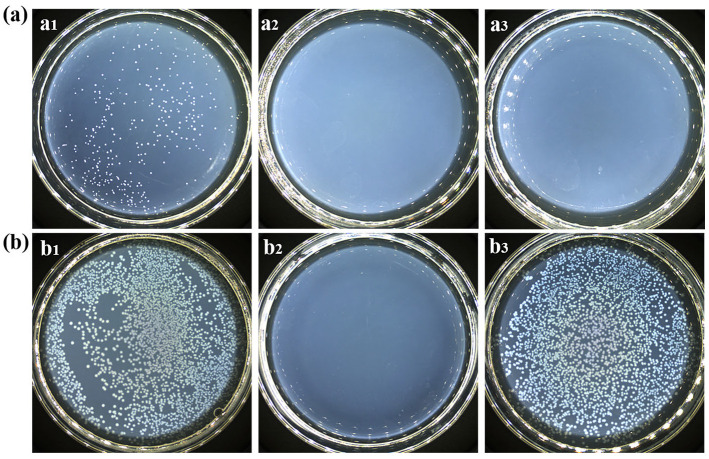
Antibacterial effects of cotton fabric samples dyed with water and ethanol extracts: (**a**) *S. aureus* and (**b**) *E. coli*: (**a1**,**b1**) undyed fabric; (**a2**,**b2**) fabrics dyed with the water extract; (**a3**,**b3**) fabrics dyed with the ethanol extract.

The absorption spectra of flavonoids are strongly pH-dependent [45]. Under conditions of pH 3–4 (Figure 4a) and 5–6 (Figure 4b), the peak shape and position of the CCS extracts remained unchanged, indicating good stability under acidic conditions. However, under highly acidic conditions (pH 3–4), some flavonoid glycosides in the extract may hydrolyze into aglycones and sugar moieties [46], which can slightly lighten the color of the extract.

Under alkaline conditions, the UV–Vis absorption spectrum of the extract exhibited a redshift, accompanied by a deepening of its color. This phenomenon can be attributed to the high degree of instability of flavonoids in alkaline solutions [46]. Jurasekova et al. observed that, under alkaline conditions, the UV–Vis absorption peak of luteolin gradually weakened with increasing pH, and a new absorption peak appeared at 398 nm, owing to the deprotonation of the –OH group on the B-ring [47]. This finding may also explain the phenomena observed in this experiment: at pH 7–8 (Figure 4c), partial dissociation of hydroxyl groups in the alkaline environment caused a subtle redshift in the UV–Vis absorption spectrum and deepening of the dye solution color. When the pH exceeded 8 (Figure 4d), the flavonoid structure was disrupted, causing the color to transition from yellow to red and purple. These results suggest that CCS extraction and dyeing under alkaline conditions should be avoided because neutral to weakly acidic conditions are more favorable for preserving CCS pigments.

### 3.5. Color Characteristics of the Dyed Fabrics

The binding pathways of CCS pigment to cotton fibers varied across pre-, meta-, and post-mordanting methods. During pre-mordanting (Figure 5a), Al^3+^ initially binds to the fibers and then coordinates with the C-3 and C-5 hydroxyl groups, as well as the dihydroxyl groups in the B ring of the dye molecules, resulting in a more reddish hue [48,49]. In the meta-mordanting process, dye molecules adsorb through two primary mechanisms: the formation of stable complexes with Al^3+^ ions and hydrogen bonding between the phenolic hydroxyl groups of flavonoids and the hydroxyl groups of the fiber. In post-mordanting, the dye penetrates the fiber; then, Al^3+^ binds to both the fiber and dye molecules. Pre-mordanted fabrics demonstrate the higher binding efficiency of CCS pigments on cotton fibers than those treated by the meta-mordanting method (Figure 5). In post-mordanting, when the ethanol concentration was below 30%, the K/S value of cotton fabric was higher than that of the other two methods, reflecting a higher interaction between the mordant and dye [50]. Notably, the post-mordanted cotton fabric with 20% ethanol showed the deepest color, achieving a K/S value of 9.87. Furthermore, the post-mordanted samples exhibited less red and yellow light. As the ethanol concentration increased further, its effect on fiber swelling became more pronounced, resulting in a significant decrease in dye uptake. This impact was particularly evident in the post-mordanting processes.

### 3.6. Color Fastness of the Dyed Fabrics

Mordanting can improve color fastness [51,52]. Cotton fabrics dyed with CCS extract using pre-, meta-, and post-mordanting methods exhibited good dry and wet rubbing fastness, achieving ratings of 4–5 (Table 5). Furthermore, the post-mordanted cotton fabrics demonstrated superior wash fastness than the meta-mordanted and pre-mordanted fabrics. In both pre-mordanting and meta-mordanting processes, Al^3+^ tended to form complexes with the dye molecules rather than bonding strongly with the fibers, reducing interaction between the dye and fiber and resulting in poorer wash fastness [53].

### 3.7. Analysis of Antibacterial Properties

The antibacterial properties of the three prepared fabric samples were evaluated (Figure 6). The cotton fabrics dyed with the water extract demonstrated excellent antibacterial properties against both *S. aureus* and *E. coli*, with a bacterial inhibition rate exceeding 99.0%. In contrast, fabrics dyed with the ethanol extract showed good activity against *S. aureus* (>99.0%) but were ineffective against *E. coli*.

Both the water and ethanol stripping solutions contained procyanidin B1 and proanthocyanidin B2, which exhibited antibacterial activity against Gram-negative bacteria [54]. Additionally, hesperetin [55] from the water stripping solution and ombuoside [56] from the ethanol stripping solution demonstrated antibacterial activity against both Gram-negative and Gram-positive bacteria. However, fabrics dyed with the ethanol extract showed no antibacterial activity against *E. coli*, which may be explained by differences in the types and concentrations of these compounds in the two fabrics. Numerous reviews on the antibacterial activity of flavonoids indicate that Gram-positive bacteria are considered more sensitive to the inhibitory effects of plants than Gram-negative bacteria [57,58,59]. This difference is attributed to the complex outer membrane of Gram-negative bacteria, which limits the penetration of flavonoid molecules [60,61]. Moreover, the lower fixation efficiency of the ethanol extract on the fabric during the dyeing process may lead to an inadequate surface concentration of bioactive compounds, which is insufficient to inhibit *E. coli*.

## 4. Conclusions

This study investigated the dissolution characteristics of CCS pigments in water–ethanol mixed solvents and their binding mechanism with cotton fibers, establishing a systematic dyeing process. The results demonstrate that water is more effective for extracting water-soluble components such as flavonoid glycosides and proanthocyanidins, while ethanol is better suited for extracting flavonols and flavonol glycosides. This finding provides a theoretical basis for solvent selection in the extraction of CCS pigments. Appropriate ethanol concentration and mordant dyeing processes can improve dyeing properties. Furthermore, it was found that cotton fabrics dyed with the water extract exhibited inhibitory effects against both *E. coli* and *v*, whereas those dyed with the ethanol extract were effective specifically against *S. aureus*. This finding provides new possibilities for the application of CCS dye in the field of functional textiles. The study not only provides solid theoretical support and practical technical pathways for the industrial application of CCS dyes but also offers new research insights and practical approaches for the green and sustainable development of natural dyes.

## Figures and Tables

**Table 1:** Common constituents in CCS water and ethanol extracts.

Number	Ingredient	Number	Ingredient	Number	Ingredient
1	Eriodictyol	8	Kaempferol-3-O-glucorhamnoside	15	Vitexin 2″-O-p-coumarate
2	Neoisoastilbin	9	Kaempferol-3-O-neohesperidoside	16	Verbenalin
3	Saponarin	10	Glucosyl-vitexin	17	Naringenin-7*-O-β-D-*glucoside
4	Taxifolin 7-rhamnoside	11	Tropine	18	Vicenin II
5	Neoastilbin	12	Proanthocyanidin B2	19	Ombuoside
6	Bilobetin	13	Vitexin-4″-O-glucoside	20	Hesperetin
7	Kaempferol 3-rutinoside	14	Procyanidin B1	21	Sanggenone H

**Table 2:** Different constituents in CCS water and ethanol extracts.

Water Extracts				Ethanol Extracts			
Number	Ingredient	Number	Ingredient	Number	Ingredient	Number	Ingredient
1	Coumarin 6	12	Kuwanon G	1	Kuwanon A	12	Kaempferol 7*-O-β*-*D*-glucoside
2	Paederoside	13	Vicenin I	2	Aromadendrin	13	Laricitrin 3-O-glucoside
3	Dracorhodin	14	Apioside	3	Isomucronulatol		
4	Schaftoside	15	5-O-Demethylnobiletin	4	Auriculasin		
5	Kurarinone, 2′-O-methyl-	16	4′-O-Glucosylvitexin	5	Quercitrin		
6	Kirenol	17	Pectolinarin	6	Quercetin 7-rhamnoside		
7	Corylifol A	18	Engeletin	7	Astragalin		
8	Polyporusterone A	19	6″-O-Malonylgenistin	8	Orientin		
9	Hinokiflavone	20	Avicularin	9	Morusin		
10	Amentoflavone	21	Plantagoside	10	Luteolin-7-*O-β*-*D*-glucoside		
11	Hesperetin 7-O-glucoside	22	Hypocrellin A	11	Homoorientin		

**Table 3:** Common constituents in CCS water and ethanol stripping solutions.

Number	Ingredient	Number	Ingredient
1	Glucosyl-vitexin	7	Kaempferol-3-O-glucorhamnoside
2	Vicenin II	8	Kaempferol 3-rutinoside
3	Vitexin 2″-O-p-coumarate	9	Proanthocyanidin B2
4	Procyanidin B1	10	Vitexin -4″-O-glucoside
5	Tropine	11	Kaempferol-3-O-neohesperidoside
6	Saponarin	12	Naringenin-7-*O-β*-D-glucoside

**Table 4:** Different constituents in CCS water and ethanol stripping solutions.

Water Stripping				Ethanol Stripping			
Ingredient	M/Z	Molecular Formula	Structural Formula	Ingredient	M/Z	Molecular Formula	Structural Formula
Bilobetin	552.11	C_31_H_20_O_10_	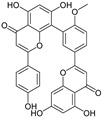	Ombuoside	638.19	C_29_H_34_O_16_	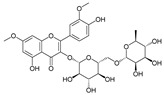
Hesperetin	302.08	C_16_H_14_ O_6_	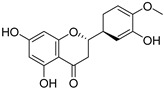				
Kirenol	338.25	C_20_H_34_O_4_	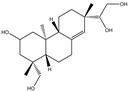				
Hypocrellin A	546.15	C_30_H_26_O_10_	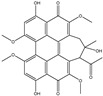				
Hinokiflavone	538.09	C_30_H_18_O_10_	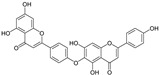				

**Table 5:** Rubbing fastness and wash fastness of cotton fabrics.

Sample	Pre-Mordanted			Meta-Mordanted			Post-Mordanted		
	Rubbing Fastness		Wash Fastness	Rubbing Fastness		Wash Fastness	Rubbing Fastness		Wash Fastness
	Dry	Wet		Dry	Wet		Dry	Wet	
0	5	5	2–3	5	4–5	3	5	4–5	2–3
10%	5	4	2–3	5	4–5	3	5	4	3
20%	5	4–5	2–3	5	4	3	4–5	4	3–4
30%	5	4	2–3	5	4	3–4	5	4–5	4–5
40%	5	4	2–3	4	4	3	5	4–5	4
50%	5	4–5	3	5	4	3	5	4–5	4
60%	5	4–5	3–4	5	4–5	3–4	5	4–5	4
70%	4–5	4–5	3–4	5	4–5	2	5	4–5	4
80%	4–5	4	2–3	5	5	2–3	5	4–5	4
90%	5	4–5	4	5	5	2–3	5	4–5	4
100%	5	4–5	3–4	5	5	2–3	5	4–5	4

## Data Availability

The original contributions presented in this study are included in the article/Supplementary Materials. Further inquiries can be directed to the corresponding author.

## Supplementary Materials

The following supporting information can be downloaded at: https://www.mdpi.com/article/10.3390/ma19040647/s1.

## References

[1] Mesrar F.E., Tachallait H., Bougrin K., Benhida R. (2024). Ultrasound-assisted extraction of vegetable dyes and mordants from wool dyed with Curcuma longa and Reseda luteola. Ind. Crops Prod..

[2] Rehman A., Ahmad A., Hameed A., Kiran S., Farooq T. (2021). Green dyeing of modified cotton fabric with Acalypha wilkesiana leave extracts. Sustain. Chem. Pharm..

[3] Repon M.R., Islam T., Islam T., Ghorab A.E., Rahman M.M. (2023). Cleaner pathway for developing bioactive textile materials using natural dyes: A review. Environ. Sci. Pollut. Res..

[4] Rahmani Z., Karimi M., Saffari I., Mirzaei H., Nejati M., Sharafati Chaleshtori R. (2024). Nanoemulsion and nanoencapsulation of a hydroethanolic extract of Nettle (Urtica dioica) and Wormwood (Artemisia absinthium): Comparison of antibacterial and anticancer activity. Front. Chem..

[5] Yang Q.-Y., Zhang T., He Y.-N., Huang S.-J., Deng X., Han L., Xie C.-G. (2020). From natural dye to herbal medicine: A systematic review of chemical constituents, pharmacological effects and clinical applications of indigo naturalis. Chin. Med..

[6] Santiago D., Cunha J., Cabral I. (2023). Chromatic and medicinal properties of six natural textile dyes: A review of eucalyptus, weld, madder, annatto, indigo and woad. Heliyon.

[7] Sun L., Feng Y., Qian X., Jin S., Liang Q. (2023). Enhancing Antibacterial Performance of Viscose Spunlaced Nonwovens by Wormwood Extract Microcapsule Finishing. Fibers Polym..

[8] Ivanov I., Vasileva A., Tasheva D., Dimitrova M. (2023). Isolation and characterization of natural inhibitors of post-proline specific peptidases from the leaves of *Cotinus coggygria* Scop. J. Ethnopharmacol..

[9] Deng Z.J., Hu X.F., Ai X.R., Yao L., Deng S.M., Pu X., Song S.Q. (2015). Dormancy release of *Cotinus coggygria* seeds under a pre-cold moist stratification: An endogenous abscisic acid/gibberellic acid and comparative proteomic analysis. New For..

[10] Miao C.-Y., Li Y., Yang J., Mao R.-L. (2017). Landscape genomics reveal that ecological character determines adaptation: A case study in smoke tree (*Cotinus coggygria* Scop.). BMC Evol. Biol..

[11] Yue X., Li G., Chen X., Li Z., Gu H., Chen H., Peng W. (2022). Nano Catalysis of Biofuels and Biochemicals from *Cotinus coggygria* Scop. Wood for Bio-Oil Raw Material. Polymers.

[12] Deniz F.S.S., Salmas R.E., Emerce E., Cankaya I.I.T., Yusufoglu H.S., Orhan I.E. (2020). Evaluation of collagenase, elastase and tyrosinase inhibitory activities of *Cotinus coggygria* Scop. through in vitro and in silico approaches. S. Afr. J. Bot..

[13] Abu-Ghosh S., Sukenik N., Amar Z., Iluz D. (2023). Yellow dyes in archaeological textiles: Sources, locations, identification, and challenges. J. Archaeol. Sci. Rep..

[14] Petroviciu I., Teodorescu I., Albu F., Virgolici M., Nagoda E., Medvedovici A. (2019). Dyes and biological sources in nineteenth to twentieth century ethnographic textiles from Transylvania, Romania. Herit. Sci..

[15] Rahman S., Jan G., Jan F.G., Rahim H.U. (2022). Phytochemical Investigation and Therapeutical Potential of *Cotinus coggygria* Scop. in Alloxan-Induced Diabetic Mice. Oxid. Med. Cell. Longev..

[16] Danjolli-Hashani D., Selen-Isbilir S. (2022). Cytotoxic effect of *Cotinus coggygria* extract on Hep3B cancer cell line. Nat. Prod. Res..

[17] Marčetić M., Božić D., Milenković M., Malešević N., Radulović S., Kovačević N. (2012). Antimicrobial, antioxidant and anti-inflammatory activity of young shoots of the smoke tree, *Cotinus coggygria* Scop. Phytother. Res..

[18] Türkmen N., Kirici S., Özgüven M., İnan M., Kaya D.A. (2004). An investigation of dye plants and their colourant substances in the eastern Mediterranean region of Turkey. Bot. J. Linn. Soc..

[19] Valianou L., Stathopoulou K., Karapanagiotis I., Magiatis P., Pavlidou E., Skaltsounis A.-L., Chryssoulakis Y. (2009). Phytochemical analysis of young fustic (*Cotinus coggygria* heartwood) and identification of isolated colourants in historical textiles. Anal. Bioanal. Chem..

[20] Sabarikirishwaran P., Unpaprom Y., Ramaraj R. (2023). Effects of Natural Dye Solvent Extraction on the Efficiency of Dye-Sensitive Solar Cells from the Leaf Biomass of Sandoricum koetjape and Syzygium samarangense. Waste Biomass Valorization.

[21] Scarano P., Prigioniero A., Tartaglia M., Zuzolo D., Maisto M., Ranauda M.A., Schicchi R., Geraci A., Sciarrillo R., Guarino C. (2024). *Rhus coriaria* L. in tradition and innovation like natural dye. Sci. Rep..

[22] Prabavathy N., Shalini S., Balasundaraprabhu R., Velauthapillai D., Prasanna S., Walke P., Muthukumarasamy N. (2017). Effect of solvents in the extraction and stability of anthocyanin from the petals of Caesalpinia pulcherrima for natural dye sensitized solar cell applications. J. Mater. Sci. Mater. Electron..

[23] Al-yaqoobi A., Hogg D., Zimmerman W.B. (2016). Microbubble Distillation for Ethanol-Water Separation. Int. J. Chem. Eng..

[24] Silva E.V., Alves J.L.F., Mumbach G.D., Reus G.F., Machado R.A.F., Bolzan A., Marangoni C. (2024). Performance comparison of falling film distillation process configurations for energy-saving ethanol-water separation. J. Ind. Eng. Chem..

[25] Çiçek Polat D., Gümüşok S., Rızvanoğlu S.S., Eryilmaz M. (2023). Bioactivities of *Cotinus coggygria* and its HPLC-DAD phenolic profiles. Plant Biosyst..

[26] Stojković D., Dragičević N., Ivanov M., Gajović N., Jurišević M., Jovanović I., Tomović M., Živković J. (2025). New Evidence for ^Cotinus coggygria^ Scop. Extracts Application in Gastrointestinal Ailments. Pharmaceuticals.

[27] Sutlović A., Glogar M.I., Čorak I., Tarbuk A. (2021). Trichromatic Vat Dyeing of Cationized Cotton. Materials.

[28] Powar A., Perwuelz A., Behary N., vinh Hoang L., Aussenac T., Loghin C., Sergiu Maier S., Guan J., Chen G. (2021). Investigation into the color stripping of the pigment printed cotton fabric using the ozone assisted process: A study on the decolorization and characterization. J. Eng. Fibers Fabr..

[29] (2016). Textiles—Tests for Colour Fastness—Part X12: Colour Fastness to Rubbing.

[30] (2010). Textiles—Tests for Colour Fastness—Part C01: Colour Fastness to Washing: Test 1.

[31] (2008). Textiles—Tests for Colour Fastness—Grey Scale for Assessing Change in Colour.

[32] (2019). Antibacterial Finishes on Textile Materials: Assessment of.

[33] Anouar E.H., Gierschner J., Duroux J.-L., Trouillas P. (2012). UV/Visible spectra of natural polyphenols: A time-dependent density functional theory study. Food Chem..

[34] Sisa M., Bonnet S.L., Ferreira D., Van der Westhuizen J.H. (2010). Photochemistry of Flavonoids. Molecules.

[35] Youssef Moustafa A.M., Khodair A.I., Saleh M.A. (2009). Isolation, structural elucidation of flavonoid constituents fromLeptadenia pyrotechnicaand evaluation of their toxicity and antitumor activity. Pharm. Biol..

[36] Benarba B., Elmallah A., Pandiella A. (2019). Bryonia dioica aqueous extract induces apoptosis and G2/M cell cycle arrest in MDA-MB 231 breast cancer cells. Mol. Med. Rep..

[37] Aguilar N., Bol-Arreba A., Atilhan M., Aparicio S. (2023). Theoretical Insights into Flavonol Solubilization by Deep Eutectic Solvents. ACS Food Sci. Technol..

[38] Rha C.-S., Kim H.G., Baek N.-I., Kim D.-O., Park C.-S. (2020). Amylosucrase from Deinococcus geothermalis can be modulated under different reaction conditions to produce novel quercetin 4′-O-α-d-isomaltoside. Enzym. Microb. Technol..

[39] Taniguchi M., LaRocca C.A., Bernat J.D., Lindsey J.S. (2023). Digital Database of Absorption Spectra of Diverse Flavonoids Enables Structural Comparisons and Quantitative Evaluations. J. Nat. Prod..

[40] Jung Y.S., Kim H.-G., Oh S.M., Lee D.Y., Park C.-S., Kim D.-O., Baek N.-I. (2023). Synthesis of Alpha-Linked Glucosides from Soybean Isoflavone Aglycones Using Amylosucrase from Deinococcus geothermalis. J. Agric. Food Chem..

[41] Han R., Ge B., Jiang M., Xu G., Dong J., Ni Y. (2017). High production of genistein diglucoside derivative using cyclodextrin glycosyltransferase from Paenibacillus macerans. J. Ind. Microbiol. Biotechnol..

[42] Dong X., Gu Z., Hang C., Ke G., Jiang L., He J. (2019). Study on the salt-free low-alkaline reactive cotton dyeing in high concentration of ethanol in volume. J. Clean. Prod..

[43] El Seoud O.A., Fidale L.C., Ruiz N., D’Almeida M.L.O., Frollini E. (2007). Cellulose swelling by protic solvents: Which properties of the biopolymer and the solvent matter?. Cellulose.

[44] Oreopoulou A., Tsimogiannis D., Oreopoulou V. (2019). Extraction of Polyphenols from Aromatic and Medicinal Plants: An Overview of the Methods and the Effect of Extraction Parameters. Polyphenols in Plants.

[45] Stavenga D.G., Leertouwer H.L., Dudek B., van der Kooi C.J. (2021). Coloration of Flowers by Flavonoids and Consequences of pH Dependent Absorption. Front. Plant Sci..

[46] Álvarez-Diduk R., Ramírez-Silva M.T., Galano A., Merkoçi A. (2013). Deprotonation Mechanism and Acidity Constants in Aqueous Solution of Flavonols: A Combined Experimental and Theoretical Study. J. Phys. Chem. B.

[47] Jurasekova Z., Domingo C., Garcia-Ramos J.V., Sanchez-Cortes S. (2014). Effect of pH on the chemical modification of quercetin and structurally related flavonoids characterized by optical (UV-visible and Raman) spectroscopy. Phys. Chem. Chem. Phys..

[48] Pękal A., Pyrzynska K. (2014). Evaluation of Aluminium Complexation Reaction for Flavonoid Content Assay. Food Anal. Methods.

[49] Jurd L. (1969). Aluminum complexes of phenolic flavones. Spectral and structural correlations. Phytochemistry.

[50] Rahayuningsih E., Fatimah W.S., Miladatussholihah N. (2025). The Influence of Mordanting Process Variables on the Cotton Fabric Dyeing with Yellow Natural Dye from Extract of *Arcangelisia flava* (L.) Merr Stem. Fibers Polym..

[51] Darmawan A., Widowati, Riyadi A., Muhtar H., Kartono, Adhy S. (2024). Enhancing cotton fabric dyeing: Optimizing Mordanting with natural dyes and citric acid. Int. J. Biol. Macromol..

[52] Raza Naqvi S.A., Ul-Wara K., Adeel S., Mia R., Hosseinnezhad M., Rather L.J., Imran M. (2024). Modern ecofriendly approach for extraction of luteolin natural dye from weld for silk fabric and wool yarn dyeing. Sustain. Chem. Pharm..

[53] Moiz A., Aleem Ahmed M., Kausar N., Ahmed K., Sohail M. (2010). Study the effect of metal ion on wool fabric dyeing with tea as natural dye. J. Saudi Chem. Soc..

[54] Mayer R., Stecher G., Wuerzner R., Silva R.C., Sultana T., Trojer L., Feuerstein I., Krieg C., Abel G., Popp M. (2008). Proanthocyanidins: Target Compounds as Antibacterial Agents. J. Agric. Food Chem..

[55] Choi S.-S., Lee S.-H., Lee K.-A. (2022). A Comparative Study of Hesperetin, Hesperidin and Hesperidin Glucoside: Antioxidant, Anti-Inflammatory, and Antibacterial Activities In Vitro. Antioxidants.

[56] Amaro-Luis J.M., Adrián M., Díaz C. (1997). Isolation, identification and antimicrobial activity of ombuoside from Ste-via triflora. Ann. Pharm. Fr..

[57] Raji Y., Nadi A., Chemchame Y., Mechnou I., Bouari A.E.L., Cherkaoui O., Zyade S. (2023). Eco-friendly Extraction of Flavonoids Dyes from Moroccan (*Reseda luteola* L.), Wool Dyeing, and Antibacterial Effectiveness. Fibers Polym..

[58] Baseri S. (2023). Agricultural crop of Scrophularia striata as a new dye for eco-friendly dyeing and bioactive finishing of handwoven piles. Sustain. Chem. Pharm..

[59] Yan Y., Xia X., Fatima A., Zhang L., Yuan G., Lian F., Wang Y. (2024). Antibacterial Activity and Mechanisms of Plant Flavonoids against Gram-Negative Bacteria Based on the Antibacterial Statistical Model. Pharmaceuticals.

[60] Yuan G., Xia X., Guan Y., Yi H., Lai S., Sun Y., Cao S. (2022). Antimicrobial Quantitative Relationship and Mechanism of Plant Flavonoids to Gram-Positive Bacteria. Pharmaceuticals.

[61] Jeanloz R.W. (1967). The chemical structure of the cell wall of Gram-positive bacteria. Pure Appl. Chem..

