# Supplementary material for: Extraction Components and Dyeing Effect of Cotinus coggygria Scop. in Water–Ethanol Systems
Source: Materials (Basel). 2026 Feb 7;19(4):647. doi: 10.3390/ma19040647 (PMC12941689; doi:10.3390/ma19040647)
Supplement: Supplementary file 1 [file materials-19-00647-s001.zip › materials-4109616-supplementary.pdf]

Table S1. Common constituents in CCS water and ethanol extracts.

Number	Ingredient	Structural formula	solubility
1	Sanggenone H	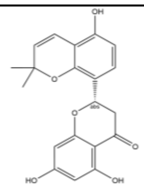	★
2	Eriodictyol	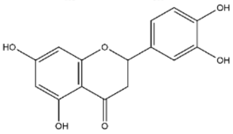	★★<br>●●
3	Taxifolin 7-rhamnoside	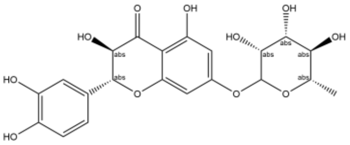	★★
4	Neoastilbin	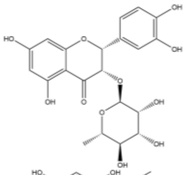	★★
5	Bilobetin	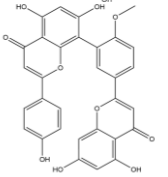	★★<br>●●●
6	Kaempferol 3-rutinoside	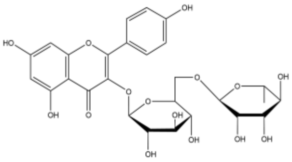	●●●
7	Vitexin 2"-O-p-coumarate	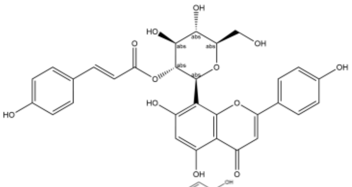	
8	Kaempferol-3-O-glucorhamnoside	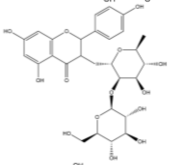	★★<br>●●●
9	Vicenin II	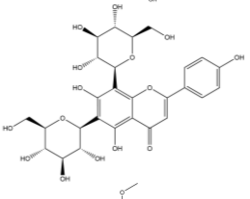	★★<br>●●●
10	Ombuoside	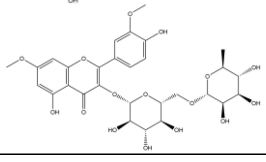	★★<br>●●●

11	Hesperetin	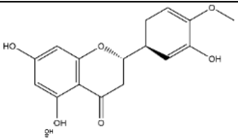	●●●
12	Glucosyl-vitexin	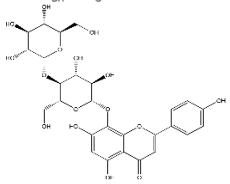	●●●
13	Naringenin-7-O-β-D-glucoside	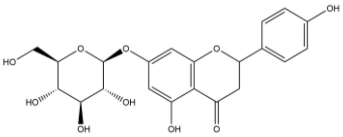	●●●
14	Verbenalin	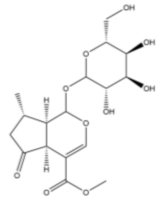	★★★ ●●
15	Vitexin -4''-O-glucoside	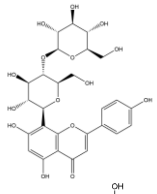	★★★ ●●●
16	Neoisoastilbin	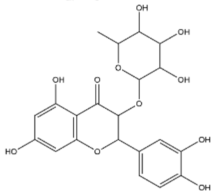	★★★ ●●●
17	Saponarin	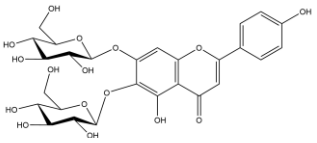	★★★ ●●●
18	Tropine	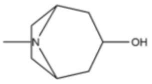	★★★ ●●●
19	Proanthocyanidin B2	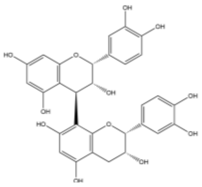	★★★ ●●●
20	Procyanidin B1	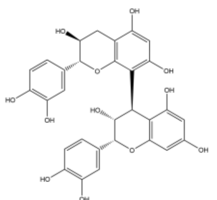	★★★ ●●●
21	Kaempferol-3-O-neohesperidoside	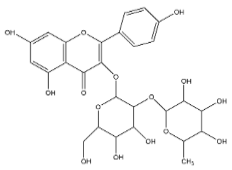	★★★ ●●●

poorly soluble in water ★; slightly soluble in water ★★; water-soluble ★★★.
poorly soluble in ethanol ●; slightly soluble in ethanol ●●; ethanol-soluble ●●●.

Table S2. Different constituents in CCS water extract.

Number	Ingredient	Structural formula	solubility
1	Coumarin 6	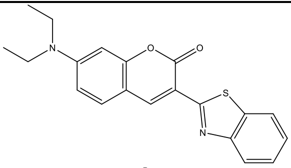	★ ●●●
2	Paederoside	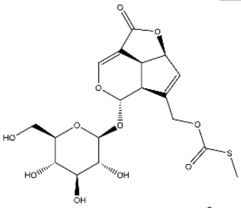	●●●
3	Dracorhodin	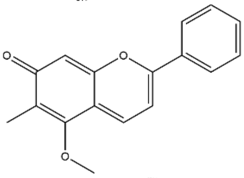	★ ●●●
4	Schaftoside	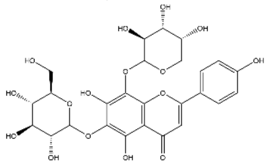	★ ●●●
5	Kurarinone, 2'-O-methyl-	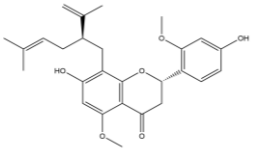	★ ●●●
6	Kirenol	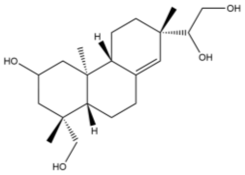	★ ●●●
7	Corylifol A	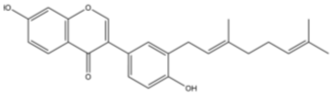	★ ●●●
8	Polyporusterone A	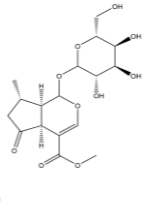	★
9	Hinokiflavone	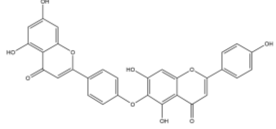	★ ●●●

10	Amentoflavone	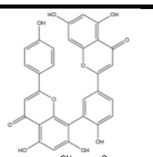	★<br>●●●
11	Hesperetin 7-O-glucoside	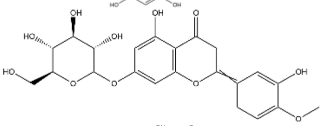	★<br>●●●
12	Kuwanon G	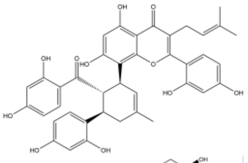	
13	Vicenin I	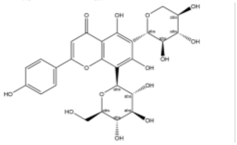	
14	Apioside	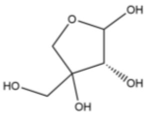	★★<br>●●●
15	5-O-Demethylnobiletin	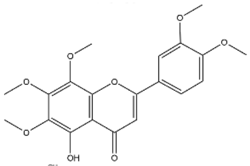	★★<br>●●●
16	4'-O-Glucosylvitexin	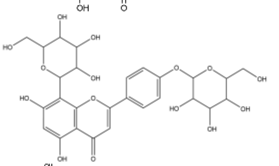	★★★<br>●●●
17	Pectolinarin	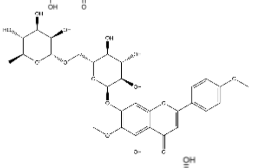	★★★<br>●●●
18	Engeletin	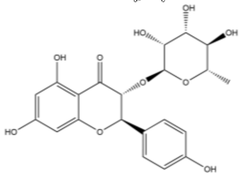	★★★<br>●●●
19	6''-O-Malonylgenistin	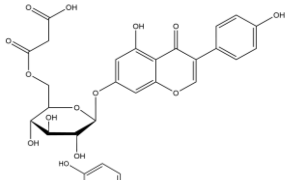	★★★<br>●●●
20	Avicularin	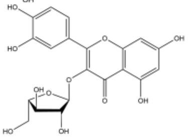	★★★<br>●●●
21	Plantagoside	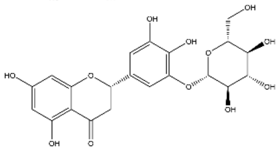	★★★<br>●●●

22	Hypocrellin A	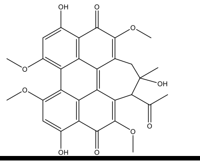	★<br>●

poorly soluble in water ★; slightly soluble in water ★★; water-soluble ★★★.
poorly soluble in ethanol ●; slightly soluble in ethanol ●●; ethanol-soluble ●●●.

Table S3. Different constituents in CCS ethanol extract.

Number	Ingredient	Structural formula	solubility
1	Kuwanon A	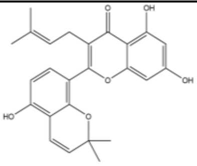	★<br>●
2	Aromadendrin	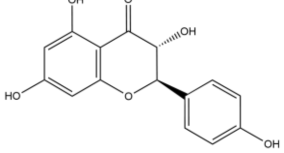	●●
3	Isomucronulatol	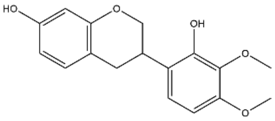	★
4	Auriculasin	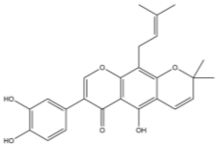	★<br>●●●
5	Quercitrin	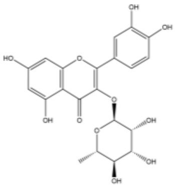	★<br>●●●
6	Quercetin 7-rhamnoside	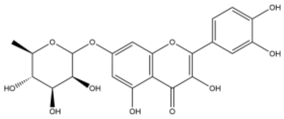	★<br>●●●
7	Astragalin	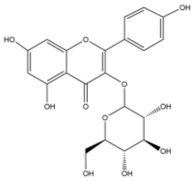	★<br>●●●<br>)
8	Orientin	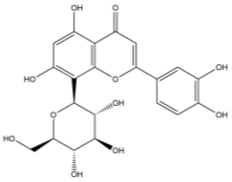	★<br>●●●

9	Morusin	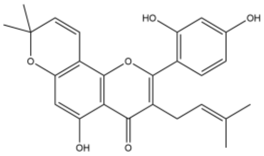	★★<br>●●●
10	Luteolin-7-O-β-D-glucoside	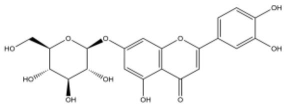	★★<br>●●●
11	Homoorientin	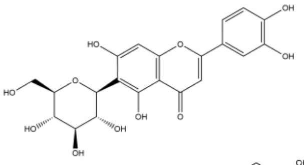	★★★<br>●●●
12	Kaempferol 7-O-β-D-glucoside	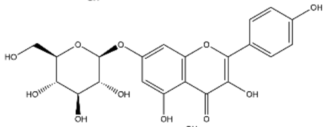	★★★<br>●●●
13	Laricitrin 3-O-glucoside	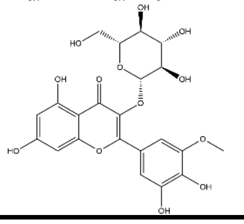	★★★

poorly soluble in water ★; slightly soluble in water ★★; water-soluble ★★★**.**
poorly soluble in ethanol ●; slightly soluble in ethanol ●●; ethanol-soluble ●●●.

Table S4. Common constituents in CCS water and ethanol stripping solutions.

Number	Ingredient	Structural formula	solubility
1	Glucosyl-vitexin	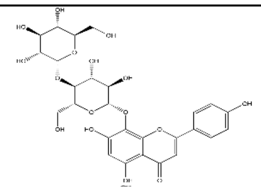	●●●
2	Vicenin II	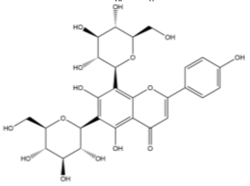	★★<br>●●●
3	Vitexin 2''-O-p-coumarate	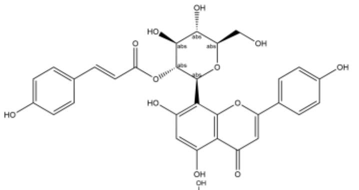	
4	Procyanidin B1	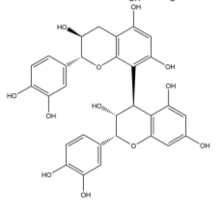	★★★<br>●●●
5	Tropine	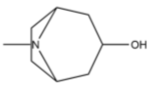	★★★<br>●●●

6	Saponarin	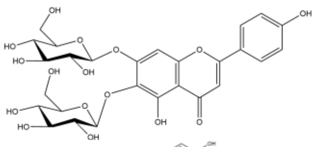	★★★<br>●●●
7	Kaempferol-3-O-glucorhamnoside	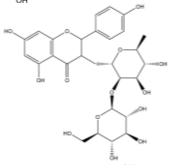	★★<br>●●●
8	Kaempferol 3-rutinoside	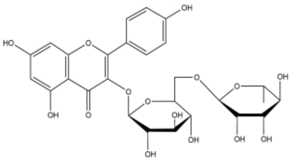	●●●
9	Proanthocyanidin B2	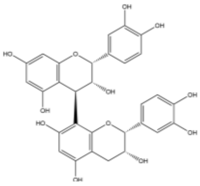	★★★<br>●●●
10	Vitexin -4''-O-glucoside	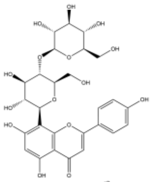	★★★<br>●●●
11	Kaempferol-3-O-neohesperidoside	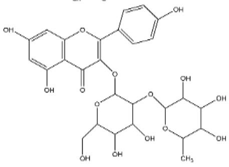	★★★<br>●●●
12	Naringenin-7-O-β-D-glucoside	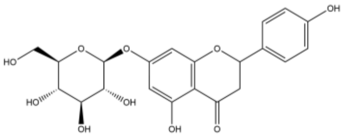	●●●

Table S5. Different constituents in CCS water and ethanol stripping solutions.

Water stripping				Ethanol stripping			
Ingredient	m/z	Molecular formula	Structural formula	Ingredient	m/z	Molecular formula	Structural formula
Bilobetin	552.11	$C_{31}H_{20}O_{10}$	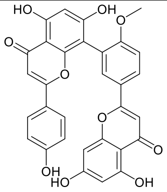	Ombuoside	638.19	$C_{29}H_{34}O_{16}$	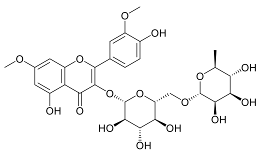
Hesperetin	302.08	$C_{16}H_{14}O_6$	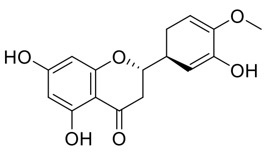				

Kirenol	338.25	$C_{20}H_{34}O_4$	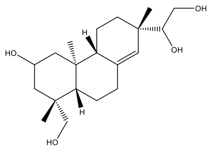
Hypocrellin A	546.15	$C_{30}H_{26}O_{10}$	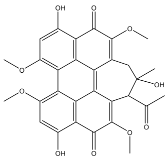
Hinokiflavone	538.09	$C_{30}H_{18}O_{10}$	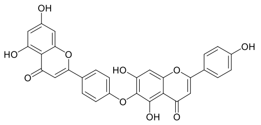
